# Direct detection and serogroup characterization of *Neisseria meningitidis* from outbreak of meningococcal meningitis in Delhi

**Published:** 2010-06

**Authors:** SS Negi, SS Grover, SS Rautela, DS Rawat, S Gupta, S Khare, S Lal, A Rai

**Affiliations:** 1 Biotechnology & Biochemistry Division; 2 Microbiology Division; 3 Directorate, National Centre for Disease Control (NCDC) (Previously NICD), 22-Shamnath marg, Delhi-54, India

**Keywords:** *Neisseria meningitidis*, *ctr A* PCR, *myn B* gene

## Abstract

**Background and Objectives:**

Rapid clinical manifestation/progression of the meningococcal meningitis and lacunae in conventional bacteriological test often encourages indiscriminate use of antibiotics much before the etiology is established. Accordingly this study was planned to evaluate *ctrA* PCR for rapid molecular detection. In addition, multiplex PCR and sequencing was done for serogroup prediction to provide essential epidemiological and laboratory evidence for decision makers of health department of the country for choosing appropriate vaccine and phylogenetic analysis to establish its lineage.

**Materials and Methods:**

73 CSF samples, collected from equal number of suspected cases, were investigated by both bacteriological (microscopy, culture, LA and drug sensitivity testing) as well as molecular tests i.e. PCR targeting conserved *ctrA* gene, multiplex PCR for serogroup characterization and DNA sequencing.

**Results:**

*ctrA* PCR revealed sensitivity, specificity, positive predictive value and negative predictive values of 93.15%, 100%,100%, and 88.23% respectively. Multiplex PCR based genogrouping followed by DNA sequencing, BLAST and phylogenetic analysis revealed complete homology with earlier submitted *Neisseria meningitidis* serogroup A strain Z2491 to suggest the sole involvement of only serogroup A in the outbreak. Two strains showed resistance to cefuroxime, ciprofloxacin, nalidixic acid. Only one strain showed resistance to ciprofloxacin, emphasizing the need for a constant surveillance system.

**Conclusion:**

These diagnostic molecular tools are of paramount importance in establishing etiology, serogrouping, and epidemiological surveillance especially in developing countries like India.

## INTRODUCTION

Meningococcal meningitis is endemic in Delhi and sporadic cases of meningococcal meningitis have occurred in the past (1, 2). In mid 2008, Delhi and its adjoining national capital region (NCR) were badly affected by sudden surge in sporadic cases of meningococcal meningitis. Meningococcal meningitis is still diagnosed mainly by clinical features and response to antibiotic treatment. However, WHO preventive strategy for such outbreak suggest early and accurate detection to initiate specific treatment and rapid implementation of mass vaccination using an appropriate polysaccharide vaccine, the success of which is based on serogroup identification (3). In view of this, urgent need for establishing etiology, serogroup prediction has been felt as conventional diagnosis based on culture and latex agglutination have several lacunae reported earlier 4–7.

Accordingly this study was done with the aims of (i) evaluating ctrA gene for rapid molecular identification of *N. meningitidis* due to its conserved nature among strains of *N. meningitidis* (ii) Drug susceptibility testing of the isolates obtained in culture (iii) Genogrouping of N.meningitidis by multiplex PCR to target orf-2 of myn B gene for serogroup A and sialyltransferase (sia D) gene for serogroup B,C, Y and W135 characterization.

## MATERIALS AND METHODS

Seventy-three CSF samples were obtained from equal number of probable meningococcal meningitis cases (as per WHO clinical case definition) admitted in different hospitals of Delhi for the period of meningococcal meningitis outbreak from March to July, 2008 (3). In addition, few Gram positive and Gram negative bacterial strains other than *N.meningitidis were listed as Citrobacter freundii, Enterobacter aerogenes, E.coli, Klebsiella pneumoniae, Proteus vulgaris, Pseudomonas aeruoginosa, Salmonella typhi, Salmonella paratyphi, Enterococcus faecalis, Staphylococcus aureus, Staphylococcus epidermidis, Streptococcus pneumoniae, Streptococcus pyogenes, Streptococcus viridians.* Fifteen CSF samples obtained from subjects suffering from conditions other than acute pyogenic meningitis (5 tuberculous meningitis, and 10 cases of viral encephalitis) were also used in the study as negative controls. The clinical isolates of *N.meninigitidis* ATCC serogroup A strain (13077), serogroup B (ATCC13090), serogroup C (ATCC13102) were obtained from ARI (Acute Respiratory Infection) laboratory of NCDC (National Centre for Disease Control).

**Microscopy.** The CSF samples were centrifuged for 20 minutes at 1500–2000 rpm and smear was prepared from the deposit without spreading the fluid. The air dried and heat fixed smear was stained by Gram staining and examined microscopically using 100X oil immersion lens. *N.meningitidis* bacilli were seen as intra and/or extracellular gram negative coffee bean shaped diplococci.

**Culture & biochemical confirmation.** The centrifuged deposit of the CSF was inoculated in chocolate and blood agar. Culture plates were incubated at 37^O^C in 5% CO_2_ atmosphere for 24–48 hours. Colonies of *N.meningitidis* appeared as small (about 1 mm in diameter), gray colored, translucent, round, and convex, bluish grey with a smooth glistening surface. Colonies were biochemically confirmed for *N.meningitidis* by testing for oxidase production (positive result) and testing for carbohydrate utilization (positive reaction for glucose, maltose and negative reaction for sucrose and lactose).

**Latex agglutination (LA).** The antigen detection in CSF was carried out by latex agglutination test using commercially available kit (LA Meningitis kit, Bio Rad, USA) following the instructions given in the kit brochure. Briefly, one drop of CSF (50 µl) after heat inactivation (100^O^C for 5 minutes) was suspended in a drop of specific latex antisera for serogroup A, B, C, D, W135, Y and mixed thoroughly. A visible agglutination within 10 minutes was recorded as positive for the specific serogroup.

**Antimicrobial susceptibility testing.** Culture isolates were subjected to drug susceptibility testing against penicillin G, amikacin, ampicillin, vancomycin, nalidixic acid, chloramphenicol, cefotaxime, ceftriaxone, imipenem, ofloxacin, moxifloxacin, cefuroxime and ciprofloxacin by E-test (MIC method) strips (AB BIODISK, Sweden) using break points recently recommended by NCCLS/CLSI (8).

**Molecular identification and serogroup characterization.** Genomic DNA was purified from 200µl of each CSF sample using QIAmp DNA Mini Kit (QIAGEN Inc., Chatsworth, Calif) as per the manufacturer's protocol.

For identification and serogroup characterization, oligonucleotide primers to specific sequences were used as described earlier (9, 10). However, thermal cycling profile was changed to certain extent from earlier studies to obtain optimal result (Table 1). For assessing sensitivity of *ctrA* PCR assay, bacterial culture in the logarithmic phase of growth (optimal density at 600 nm 0.7 to 0.8) was diluted in phosphate buffered saline. Each two fold dilution was tested in PCR assays to determine the minimal number of colony forming units (cfu) that could be detected. CFU was estimated by standard plating procedure (11, 12).

**Table 1:** Oligonucleotides primers used in this study.

Purpose	Gene target	Oligonucleotide primer sequence	PCR component concentration	Thermal cycling	Amplicon size in 2% Agarose gel
Identification	*ctrA*	5'ccagcggtattgtttggtggt3’ 5'caggcggcctttaataatttc3’	10 pmole each primer, 2.5mM MgCl2,1XPCRbuffer,200µM deoxynucleoside triphosphate, 1.25 U Taq polymerase (PerkinElmer)	950C /4min, 35cycle 950C/10 sec,550C 30sec and1 cycle at 720C/ 7min	177 bp

Serogroup characterization by multiplex PCR	Orf-2of *myn B* gene for Serogroup A	5'cgcaataggtgtatatattcttcc3’ 5'cgtaatagtttcgtatgccttctt3’	0.3 µM each primer,5mM MgCl2, 1X PCR buffer, 200µM,1U Taq polymerase (Perkin Elmer)	1cycle(940C/3min,550C/30sec,720C),35 cycle (920C/40sec,550C/30sec,720/20sec),1cycle (720C/10min)	400bp
	*SiaD* (serogroup B)	5'cgtaatagtttcgtatgccttctt3’ 5'gcatgctggaggaataagcattaa3’			450bp
	*SiaD* (serogroup C)	5'gcatgctggaggaataagcattaa3’ 5'tcaaatgagtttgcgaatagaaggt3’			250bp
	*SiaD* (serogroup W135)	5'cagaaagtgagggatttccata3’ cacaaccattttcattatagttactgt3’			120bp
	*Sia D* (Y)	5'ctcaaagcgaaggctttggtta3’ ctgaagcgttttcattataattgctaa3’			120bp

**DNA Sequencing.** PCR myn B gene amplicon's products were purified by precipitation with 3 M sodium acetate and isopropanol. Then, automated nucleotide sequencing was performed using Big Dye-Terminator Cycle Sequencing kit (Applied Biosystem, USA) using 10 to 50 ng of PCR product and 2–3 pmol of sequencing primer. PCR amplicons were sequenced using both forward and reverse primers. Extension products were purified by 3M sodium acetate and ethanol precipitation. Purified extension products were resuspended in 12 µl of Hi di formamide and vortexed for few seconds. Then, they were kept at 96^o^C for 3 min and subjected to rapid chilling by keeping on ice for 15 min before loading onto a capillary DNA sequencer (ABI 310 Genetic Analyzer, Applied Biosystem, USA). Sequences were assembled with the auto assembler DNA sequence software version 2.0, and consensus sequences were obtained using Seqed DNA software (ABI). Consensus sequences representative of each variant were then analyzed with the Gen Bank BLASTN search. Multiple sequence alignments (MSA) were done using CLUSTAL W. Phylogenetic analysis was performed by MEGA 4 software using reference sequence which were previously reported and submitted in gene data bank (AF019760.1, AL157959.1, AY234202.1, AY234203.1, AY234204.1, AY234205.1,AY281049.1, NC003116.1).

Statistical analysis was done by chi square (χ^2^) test (13).

## RESULTS

All 15 CSF samples of nonmeningococcal origin were found negative by all four tests discussed depicting 100% specificity for smear microscopy, culture, LA and *ctrA* PCR. *ctrA* PCR was also found negative in a battery of bacterial species other than *N. meningitidis* showing its high specificity.

**Fig. 1:**
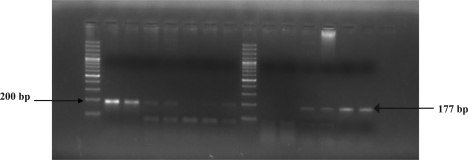
Amplified product of 177bp of *ctrA* gene from meningococcal meningitis suspected cases.

Smear microscopy, culture and LA test showed the sensitivity of 15.06% (11/73), 6.8% (5/73) and 86.30% (63/73) respectively. In comparison, the *ctrA* PCR test (Fig. 1) with sensitivity of 93.15% (68/73) was found to be much more sensitive than smear examination, culture and LA test on applying chi square test (χ^2^) at 95% confidence level (p<0.05). PCR revealed a sensitivity of less than 5 cfu. All the culture isolates obtained were confirmed as *N. meningitidis* with the biochemical tests mentioned. We compared the sensitivity of PCR test vis a vis three different tests i.e., smear examination, culture and LA test individually as well as in combination (Table 2).

**Table 2:** Sensitivity of *ctr A* PCR test with regard to three different tests, individually as well as in combination.

Test/Result category (No.)	PCR Result		Sensitivity of PCR test (%)

	positive	negative	
Smear microscopy positive (11)	11	0	100%
Smear microscopy negative (62)	50	12	80.64%
Culture positive (5)	5	0	100%
Culture negative (68)	56	12	82.35%
LA positive (63)	61	2	96.82%
LA negative (10)	5	5	50.0%
Smear and/or LA+ve but culture–ve (58)	56	2	96.5%
Smear or LA or culture+ve (63)	61	2	96.82%
Smear, culture& LA negative (10)	5	5	50.0%

The sensitivity of detection of *N. meningitidis* in smear positive samples by *ctrA* PCR approached 100%, whereas even in smear negative specimens, it had a sensitivity of 80.64%. 100% sensitivity of *ctrA* PCR was observed for culture positive samples, whereas in culture negative samples, 82.35% of sensitivity was observed. In LA test positive samples, 61 samples were found positive by *ctrA* PCR test with sensitivity of 96.82%. In LA test negative samples, 50% of samples showed positive PCR result. Among 58 smear or LA positive but culture negative samples, 56 samples were found positive by *ctrA* PCR test with sensitivity of 96.5%. In 5 out of 10 CSF samples, which were found negative by conventional test used, only *ctrA* PCR gave the confirmatory result. Positive and negative predictive value of *ctrA* PCR was recorded as 100% and 88.23% respectively.

Two culture isolates showed susceptibility to all commonly used drugs i.e. penicillin G, amikacin, ampicillin, vancomycin, nalidixic acid, chloram-phenicol, ciprofloxacin, cefotaxime, ceftriaxone, imipenem, ofloxacin, moxifloxacin and cefuroxime. However, three isolates showed resistance to one or more antibiotic. Two strains showed resistance to cefuroxime (MIC 16–17.4 µg/mL), ciprofloxacin (MIC 4.5–6 µg/mL), nalidixic acid (MIC 32–48 µg/mL), and one strain showed resistance to ciprofloxacin (MIC 6 µg/mL) only.

**Fig. 2:**
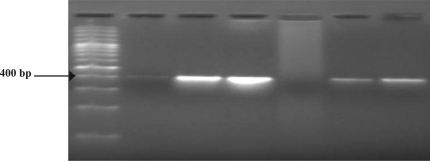
Multiplex PCR showing amplification of 400bp region of orf-2 of *myn* B gene specific for serogroup A from clinical samples of CSF. Lane 5 from left is the negative control (ddH_2_O). 100bp size marker (Perkin Elmer) are indicated in base pair at the left. Electrophoresis was done on a 2% agarose gel.

Multiplex PCR correctly characterized the serogroup of all *N. meningitidis* positive CSF samples found by *ctrA* PCR (Fig. 2). Multiplex PCR results of serogroup determination was found in accordance with the result of LA test. All strains were found belonging to serogroup A, giving 400bp amplicon specific for orf-2 of *myn* B gene characterizing serogroup A, to support the fact of prevalence of serogroup A in India. In Phylogenetic analysis using MEGA 4 software, all sequences showed similar lineage pattern corresponding to single node origin with maximum match with earlier submitted sequence of *N. meningitidis* serogroup A strain Z2491.

## DISCUSSION

During the 2008 meningococcal meningitis outbreak, similar to which Delhi had witnessed at regular intervals in the past, the need for rapid and accurate molecular diagnostic test was felt for identification and serogroup characterization to initiate specific chemotherapy and reduce morbidity and mortality. This would also provide substantial information to help decision makers of the health department of the country to use best epidemiological and laboratory evidence of serogroup involved in the outbreak for choosing the most appropriate vaccine (1, 2, 14). Laboratory based surveillance is key to efficient control of this problem. The laboratory diagnosis of meningoccal disease is usually carried out by conventional methods which lack sensitivity specially in antibiotic treated subjects. The majority of notified cases of meningococcal disease in India are based only on clinical grounds without confirmation of the etiology by a laboratory technique. In this study, we have standardized a single tube, single primer set assay developed for the rapid detection of *N. meningitidis* in CSF samples within 3 hours of sample received. Rapid turnaround time with high positive and negative predictive value of *ctrA* PCR may provide clinician the vital diagnostic information for early and effective treatment of patient to curb the spreading of the disease at initial stages.

The conserved *ctrA* gene was chosen for the standardization & evaluation of PCR assay specific for *N. meningitidis* detection. This gene codes for conserved outer membrane protein of *N. meningitidis* believed to be an ABC (ATP-binding cassette) transporter (9). *ctrA* gene was earlier demonstrated to occurs exclusively in *N. meningitidis* and not in other pathogenic or nonpathogenic Neisseria species, thus providing a good candidate for a *N. meningitidis* specific DNA based assay (15).

Optimal PCR results were obtained with a slight modification in thermal cycling parameter used earlier by Lansac *et al*. (2000), by giving 35 cycles and denaturation time of 95^o^C for 10 sec. which gave the optimal result in our study. Lansac *et al*. obtained optimal results with 30 cycles and denaturation time of 95^o^C for 1 sec. The justified reason could be lab to lab variation which were also supported by earlier studies (9).

As evident from the results, smear microscopy and culture lacked sensitivity for detection of *N. meningitidis* in clinical samples. ctrA PCR test with an overall sensitivity of 93.15% showed a significant difference over conventional tests in detection of *N. meningitidis* in the clinical samples of CSF (p<0.05). However, unusual low sensitivity in culture could be attributed most probably to fear psychosis associated with the disease causing the antibiotic treatment to be commenced as soon as possible to prevent the mortality and also some administrative delay in sending CSF samples to NCDC for laboratory testing. So, *ctrA* PCR would further be useful in outbreak circumstances where prior antibiotic treatment is inevitable. *ctrA* PCR detected *N. meningitidis* in all culture proven cases and in 56/58 samples in smear or antigen detection (LA) positive cases. The failure of *ctrA* PCR in two LA proven cases could probably be due to the presence of inhibitory substances in the specimen. Our *ctrA* PCR result showed the sensitivity, specificity, positive and negative predictive value of 93.15, 100, 100 and 88.23% which were comparable with earlier studies of Chakrabarti *et al*. (2009) showing 79.24, 97.6, 89.36, 94.88, Failace *et al*. (2005) showing 88.1, 99, 98.7, 90.1, Richardson *et al*. 2003 showing 97, 99.6, 99.6, 97%, Baethgen *et al.* (2003), showing 88.2, 100, 100, 79.4%, Taha *et al*. 2000 showing 93, 96, 98, 86% sensitivity, specificity, positive and negative predictive value respectively (4, 7, 10, 16, 17). Pedro *et al.* 2007 showed 96% sensitivity of their PCR in diagnosis of meningococcal meningitis in Brazil (6). Filippis *et al.* (2005) showed 100% sensitivity and 78.4% specificity (5). In 2005 study of Taha *et al*., 11 meningococcus reference centre located in 11 different European countries showed mean sensitivity and specificity of 89.7 and 92.7% by PCR (18). Major advantage of *ctrA* PCR over conventional test has been seen in samples undiagnosed by conventional test as from a total of 10 samples declared negative by all conventional test, 5 were found positive for *N. meningitidis* by *ctrA* PCR only with sensitivity of 50% in such cases. This percentage was found higher than that of Taha *et al*. whose studies found 35% positivity and 31% obtained by New combe *et al*. 1996 using PCR in suspected cases of meningococcal disease (10, 19).

Various other target genes were also used in the earlier studies. A PCR assay targeting the meningococcal insertion sequence IS1106 was shown to provide a rapid and sensitive assay for the diagnosis of meningococcal meningitis in patients with negative findings in culture tests due to prior antibiotic treatment. However false positive diagnosis of meningococcal infection by the IS1106 PCR has been reported with probable reason of their inherent genetic mobility of insertion sequences which lead to their transfer among bacterial species and genera (16, 20). PCR targeting 16S and/or 23S ribosomal RNA genes showed sensitivity and specificity varied from 80 to 95% and the detection limit in agarose gels ranged from 30 to 3 CFUs per reaction tube (21–23).The sensitivity level achieved with our *ctrA* PCR assay are less than 5 CFUs.

Protocols based on real time PCR have been used for simultaneous detection of *N. meningitidis, H. influenzae and Streptococcus pneumoniae* using *ctrA, bexA* and *ply* gene targets, respectively (24). This methodology promises to be the ultimate PCR diagnosis, but it is still too expensive to be applied in a public health laboratory.

Though the three isolates showed resistance to one or other drugs, their result could not corroborate any drug resistant pattern in the community. However, the result emphasizes the need of constant surveillance system to monitor the emergence and spread of antimicrobial drug resistant strains.

Genogrouping of *N. meningitidis* has been of great epidemiological importance. Genogrouping by multiplex PCR found that all *ctrA* PCR positive findings have amplified 400bp orf-2 region of *myn* B gene, confirming the sole involvement of serogroup A in this epidemic and compounding the fact that serogroup A strains are responsible for major epidemic and pandemics of meningococcal disease. Multiplex PCR should have a great implication in opening new possibilities of more reliable surveillance over conventional technique based surveillance. DNA sequencing, comparative BLAST result and phylogenetic analysis using MEGA 4 revealed complete homology of meningitidis strains involved in this outbreak with earlier submitted sequence of *N.meningitidis* serogroup A strain Z2491 to suggest the sole involvement of only serogroup A in this outbreak. We conclude by saying that these two steps approach (identification of *N.meningitidis* followed by genogrouping) seems to provide rapid identification of etiological agent and characterizing the serogroup, leading to fast track management of meningococcal outbreak situations where administering antibiotic treatment is a prerequisite condition for the clinician for controlling mortality and morbidity which often led culture techniques less sensitive for its diagnosis.
